# Physiological Basis of the Couvade Syndrome and Peripartum Onset of Bipolar Disorder in a Man: A Case Report and a Brief Review of the Literature

**DOI:** 10.3389/fpsyt.2018.00509

**Published:** 2018-10-18

**Authors:** Evangelia Giourou, Maria Skokou, Stuart Peter Andrew, Philippos Gourzis

**Affiliations:** ^1^Department of Psychiatry, School of Medicine, University of Patras, Patras, Greece; ^2^Specialist Care Team Limited, Morecambe, United Kingdom

**Keywords:** peripartum-onset, bipolar disorder, couvade syndrome, male, mood episode

## Abstract

Rapid hormonal changes during pregnancy as well as psycho-social stressors accompanying parenthood have often been associated with peripartum mood episodes in women with bipolar disorder or with not yet clinically expressed bipolar diathesis. Yet, little is known about the correlation of peripartum onset of bipolar disorder in men. We present the case of a man with bipolar disorder with peripartum onset and subsequent episodes following the peripartum initiation of the disease, as well as the association of the couvade syndrome, as a pathological response to a man due to hormonal shifts observed in males cohabiting with a pregnant female. The patient had his first depressive episode during the peripartum period of his spouse, followed by two mixed episodes with psychotic features that leaded to his compulsory psychiatric evaluation and subsequent hospitalization and the diagnosis of Bipolar Disorder I. There is a well-known correlation between the peripartum period and mood disturbances to the point of inducing full blown episodes, suggesting of a bipolar disorder initiation or mood episodes relapsing in female patients already diagnosed with bipolar disorder. Due to the patient's psychological disturbances and the phenomenology of his symptoms, mainly concerning the psychotic features accompanying his episodes, we discuss the possible underlying biological correlates as a triggering mechanism, that might overlap the manifestation of the Couvade Syndrome as well as the initiation or relapse of Bipolar Disorder in males. It seems that males are not less influenced by hormonal and psycho-social factors posed upon them during the peripartum period of their cohabiting female spouse.

## Introduction

There is a noted association between the postpartum and mood disturbances (1–3). In women, this corresponds to the rapid hormonal changes contributing to vulnerability to depression (3, 4) as well as the role of major psychosocial stressors triggering reproducing cycle-associated mood symptoms in women with often missed postpartum bipolarity (5, 6).

Women with a bipolar diathesis are more prone of having a peripartum onset of disease (i.e., during pregnancy or within 4 weeks after delivery) while there is a high risk of clinically confirmed mood episodes relapsing during postpartum period (7–9). Furthermore, misdiagnosis of bipolar disorder as major depressive disorder during the postpartum period is common, while many of these patients will often be re-diagnosed with bipolar disorder with relapsing mood episodes (8, 10). In contrast to women, the literature on the prevalence of bipolar disorder onset or relapse in men who just became fathers is poor, even if it has been described that male postpartum depression affects 1 in 10 new fathers, comparing to the 5% of men in the general population (11).

Here, we present the case of a man with bipolar disorder with peripartum onset who had his first depressive episode immediately after becoming a father followed by two manic episodes with psychotic symptoms and mixed features that caused severe disturbance causing impairment in functioning and necessitating hospitalization.

We also discuss the association of the couvade syndrome (12, 13) as a pathological response in a male partner cohabitating with a pregnant female due to the hormonal shifts in the male (14, 15), as well as the psychosocial stressors induced on new parents regardless of their gender. The couvade syndrome can manifest with symptoms ranging from mood disturbances to a full mood episode precipitating the onset of bipolar disorder with subsequent psychotic decompensation (1–3).

## Case presentation

A 39-years-old man was compulsory admitted to our inpatient psychiatric unit because of an episode with mixed mood features and psychotic symptoms, with it being the third episode in his personal history of Bipolar Disorder with peripartum onset following the birth of his child.

He had no history of prior psychoactive substance use or encephalitis. His past medical history was free of any chronic medical disorders. Dysfunctional personality traits were described in the patient since his early adult life, such as rigidity, stubbornness, suspiciousness, hostility, and being argumentative in his interpersonal relations, indicative of premorbid paranoid personality disorder, according to DSM-5 criteria, as well as narcissistic personality disorder traits, namely lack of empathy, exploitative behavior toward his relationships, and excessive need to be admired.

He had no family psychiatric history. His mother was described as the dominant family figure with his father being withdrawn without evidence of suffering from any mood or psychotic disorder.

The patient had his first episode, being a depressive episode with onset during his wife's postpartum period, for which he received venlafaxine up to 300 mg per day which he discontinued 1 month after its initiation.

One year after his initial depressive episode, he relapsed with a manic episode, the second in his personal history, characterized by irritability, dysphoric mood, distractibility, aggressiveness, grandiosity, psychomotor agitation, increase in goal-directed activity, mood lability, decreased need of sleep, recurrent suicidal ideation, feelings of despair, and diminished pleasure in most of his activities. A mixture of mood-congruent and mood-incongruent psychotic symptoms was also present, consisting mostly of persecutory ideas, ideas of reference, grandiose ideas involving his 15 months old son, as well as regression and feelings of jealousy toward the child. The theme of his delusional ideas related to his son led the patient to inappropriate (i.e., he insisted being present during his wife's breastfeeding sessions while he demanded that she caress his hair as he leaned on her breasts near to the breastfeeding child) and dangerous behaviors toward the infant, such as bathing him in ice cold water to the point of hypothermia rationalizing the incident as an effort to “toughen up” his child. Because of this abusive and potentially dangerous behavior, compulsory psychiatric evaluation and subsequent hospitalization were ordered.

Six hundred micrograms of carbamazepine was introduced daily and the sequential add-on of olanzapine up to 20 mg per day yielded full symptomatic remission. Olanzapine was subsequently lowered to 10 mg/day and the patient was discharged with a diagnosis of Bipolar Disorder I and Paranoid Personality Disorder, returning to the premorbid level of relational and personal functioning for the following 6 months.

Six months after his first hospitalization, the patient discontinued medication and relapsed with the episode discussed, presenting with mixed mood features which resulted in a second compulsory hospitalization.

The episode was characterized by behavioral problems, aggravation of his paranoid ideation, dysphoric and irritable mood, observed distractibility, insomnia, psychomotor agitation, feelings of despair and helplessness, involvement in quarrels and aggressive behavior, withdrawal from intimate relationships, and a waste of money and assets. The physical and neurological examination, as well as the imaging and laboratory testing, did not reveal any further pathological findings since his first hospitalization.

Physical and neurological examination revealed no pathological findings while electroencephalography, thyroid, and liver function tests, electrolytes, urine and creatinine, vitamin B12 and folic acid levels, and whole blood count were all within normal levels. HIV, HBV, HCV were all negative. Chest Ro and ECG revealed no pathological findings.

A brain computerized tomography (CT) scan revealed a small hypodense wedge-shaped lesion at the left posterior parietal lobe. The patient had a history of a traumatic brain injury at the age of 15 years. The imaging finding based on its localization and features and according to neurosurgical and neurological assessments yielded no evidence of any clinical relevance to his symptoms nor had any clinical significance.

The further neurocognitive assessment did not show any alterations in the premorbid level of cognitive function.

During the patient's last hospitalization, valproate slow release was initiated up to 1,500 mg/day with the consequent add-on of olanzapine up to 15 mg/day leading to the clinical remission and discharge of the patient who recovered to his premorbid level of functioning. He also received long-term psychodynamically-oriented psychotherapy, aiming at resolving paternity issues and related conflicts.

## Discussion

This paper reports a case of a man with Bipolar Disorder I with peripartum onset, that begins within 1 month following the delivery of his son with a depressive episode, followed by two more episodes with the last presenting with mixed mood disorder symptoms with psychotic features. Besides the clinical manifestation of mood and psychotic symptoms the patient experienced during his successive mood episodes, he also manifested psychological disturbances regarding the paternal role. These were characterized by his desire to be nourished as a child himself, pointing to regression and indicative of profound immaturity and ambivalence toward fatherhood, possibly related to premorbid narcissistic personality traits being present.

Up to now, there is only one case report of bipolar disorder postpartum relapse in a male(4), two reports of postpartum psychosis (1, 3), and several reports of postpartum depression (5–7) in males. Psychosocial stressors coming with parenthood affect men as well as women, as they try to adjust to their new life's dynamics. Lack of sleep, relationship stress and spousal conflicts, childcare stress, low socioeconomic status, poor social support, non-standard family or unplanned/unwanted pregnancy, and the sense of being excluded from the connection between the mother and the child are all factors that have been associated with postpartum depression to fathers (4, 8).

However, the association between bipolar onset during the peripartum period in males has not been described. For females, it has been speculated that physicians often mistake the symptoms that indicate bipolarity during a mood episode at the postpartum such as diminished sleep, increased activity, and mood lability as normal experiences of childbirth thus overdiagnosing postpartum depression with the result of missing a possible bipolar onset of disease.

The couvade syndrome or in some cases shown as a ethno-anthropological phenomenon, a range of symptoms presented in men during their partner's pregnancies, that can take the form of ritualistic behavior seen in the male voluntary presenting a collection of social pregnancy behaviors, known as the Couvade Ritual, or the full Couvade syndrome as the occurrence in the male mate of physical symptoms related to pregnancy such as nausea and abdominal pain (9–13).

Several investigators have reported that the Couvade syndrome is related to various psychosocial factors such as anxiety in expectant fathers; empathy to total identification with the expectant mother as a somatic expression of anxiety; ambivalence about fatherhood and symbolic representation of deeper conflicts; the view of the fetus as a rival for the mother's attention and a regressive manifestation of the narcissistic injury of losing his position as a “favorite;” paternity issues; roots of fatherliness that develops secondarily to motherliness from the biological dependence on the mother to the biological sexuality as sources of fatherhood; sexuality and gender identity issues as an activation of a passive femininity; parturition envy as the male's envy of the female's ability to bear and give birth to children; defense against aggressive impulses as a self-inflicted punishment for his feelings of aggression toward the unborn child; all mostly psychodynamic in nature (12).

However, there is a strong biological perspective on the syndrome and on how it can manifest as a triggering factor or even an expression of an Axis I disorder such as a depressive disorder or bipolar disorder.

There is evidence suggesting that these shifts in behavior in males are mediated by physiological changes similar to those seen in pregnant females that induce parental care. Increases of prolactin and decreases of testosterone are associated with paternal behavior. Also, estradiol levels in men peak in the late prenatal period while cortisol levels increase immediately before birth in both men and women with up to a 75% increase from baseline (10).

Several studies have discussed the effect of prolactin (PRL) levels in bipolar disorder with a long-term Lithium treatment (>6 months) leading to lower PRL release in euthymic bipolar patients compared to the controls (14, 15) and the increased prolactin concentrations in patients in relapse (16). The baseline PRL levels in the patient of the case discussed were not elevated after the initiation of antipsychotic medication administered to control the mentioned episodes.

Heightened levels of morning cortisol in schizophrenia and bipolar disorder suggests a pathology of the hypothalamic-pituitary-adrenal (HPA) axis that may reflect a shared process of illness development in line with current stress-vulnerability models (17), while antipsychotics are a significant contributing factor to the blunted cortisol stress response in euthymic bipolar disorder patients (18). Hair cortisol concentration has been found higher in bipolar patients compared to schizophrenia patients and controls and higher in inpatients on admission than in outpatients in remission, while manic symptoms are correlated with hair cortisol concentration in bipolar and psychotic patients (19, 20).

Evaluating the possible relation between serum estrogen levels and bipolar disorder it has been described that women with postpartum psychosis show very low levels of estrogen and a significant improvement of symptoms after treatment with estrogen (21). Furthermore, studies on the selective estrogen receptor modulator tamoxifen found that tamoxifen was effective in producing antimanic effects (21). The steep fall in the levels of estrogens found in both males and females during the postpartum period and the discussed findings are indicative of postpartum being a hormonally vulnerable period for inducing manic and psychotic symptoms in patients with bipolar diathesis.

A recent case report describes the effects of testosterone therapy on an 18-years-old man with Klinefelter syndrome and bipolar disorder unresponsive to the usual pharmacotherapy such as mood stabilizers and antipsychotics in controlling his rapidly repeating relapses of manic episodes, who was improved and had no manic relapse for 3 years after initiating testosterone therapy (22). Further studies of the role of testosterone in the neurobiology of mood disorders are needed.

## Concluding remarks

In conclusion, it seems that males are not less influenced by hormonal and psychosocial factors posed on parenthood during the peripartum period. The correlation of the peripartum period and mood disturbances as well as the initiation or relapse of mood episodes in females with bipolar disorder or not yet clinically expressed bipolar diathesis, has been described in the literature, yet more studies are needed to clarify the exact biological analogs underlying the association of mood disorders with the peripartum period regardless of patients' gender, especially in fathers with an underlying bipolar diathesis or an already diagnosis of bipolar disorder, during the peripartum period of their cohabiting female spouse.

## Ethics statement

This study was performed in accordance with the provisions of the Declaration of Helsinki 2013. We obtained informed written consent from the patient authorizing publication of the clinical case. His anonymity has been preserved.

## Author contributions

EG and MS observed the patient, collected, and analyzed the data. EG, MS, SA, and PG wrote the paper. All authors approved the final work.

### Conflict of interest statement

The authors declare that the research was conducted in the absence of any commercial or financial relationships that could be construed as a potential conflict of interest.
